# How do anthropogenic changes affect wildlife microbiomes?

**DOI:** 10.1371/journal.pbio.3003939

**Published:** 2026-08-26

**Authors:** Katherine M. Lagerstrom, Andrew P. Dobson, Shane Campbell-Staton, Andrew H. Moeller

**Affiliations:** Department of Ecology and Evolutionary Biology, Princeton University, Princeton, New Jersey, United States of America

## Abstract

Human activities are transforming ecosystems, but their effects on wildlife microbiota remain poorly understood. Pollution, habitat transformation, and altered host contact networks can reshape host-associated microbiota in context-dependent ways, including through symbiont loss, selection for antimicrobial resistance, shifts toward human-associated community states, and increased among-host variability. Whether these changes are transient or persistent, harmful or adaptive, or consequential for wildlife health and disease risk is largely unknown. This Unsolved Mystery highlights key mechanistic gaps and outlines future research strategies for the field, including studies that distinguish environmental filtering, dispersal limitation, host physiological responses, and cross-species microbial transmission.

## Abstract 2

Human activities are transforming ecosystems, but their effects on wildlife microbiota remain poorly understood. This Unsolved Mystery looks at how pollution, habitat transformation, and altered host contact networks can reshape host-associated microbiota and outlines future research strategies for the field.

## Introduction

Biodiversity loss, climate change, human population expansion, resource depletion, widespread environmental pollution, and rising zoonotic disease risk are among the defining features of human impacts on the Earth in the 21st century. The rapid, human-induced environmental change associated with these pressures can negatively affect wildlife species through direct effects on their populations, but also indirectly through disruption of host–microbe interactions. Most animals harbor microbial communities (microbiota) [1], which have important roles in development, metabolism, and immunity. In mammals, birds, fish, and a diverse range of invertebrate species, the microbiota promote the training and development of the host’s immune system [2], expand the metabolic capabilities available to their host [3,4], and provide protection against pathogen invasion [5]. In some host clades, microbial strains have diversified in parallel with their hosts [6–9], reflecting long-standing symbioses maintained over evolutionary timescales. Accordingly, disruption of these symbioses, whether they be mutualistic, commensal, or parasitic, can have consequences for hosts. In humans, mice, and other model organisms, experimental manipulation of the microbiota and host–microbe interactions have been linked to altered host-level traits relevant to health and fitness, including immune dysfunction, metabolic disease, and reduced resistance to pathogen colonization [2,5,10]. Recent work has shown that anthropogenic stressors are altering host-associated microbiota in a diverse range of wildlife species (S1 and S2 Tables), raising the possibility that microbiota changes may be one mechanism linking environmental change to wildlife health and fitness. Given these potential consequences for wildlife, there is a growing need to document and synthesize the main ways in which humans are affecting these critical symbioses.

**Fig 1:**
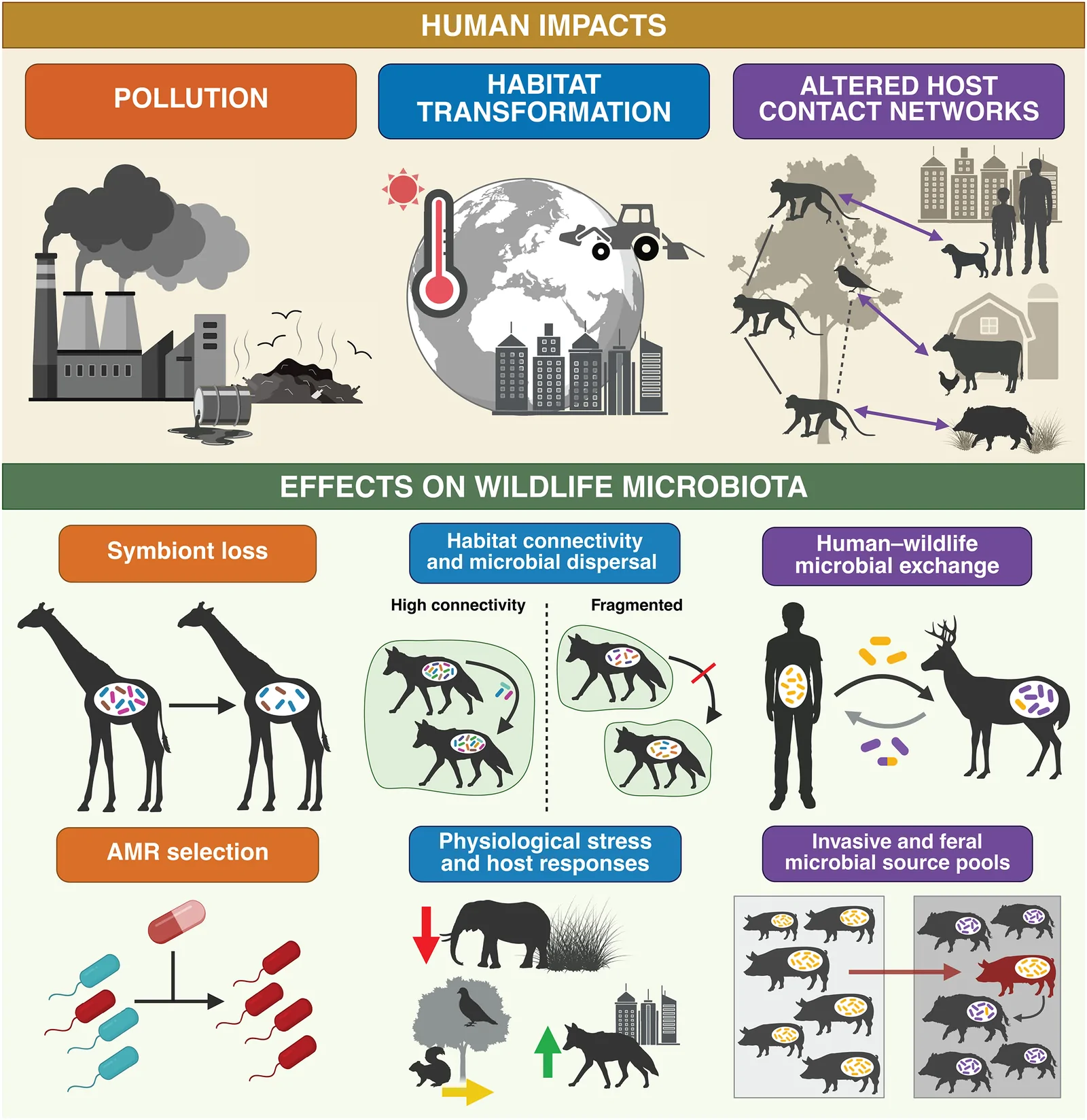
Human impacts on wildlife microbiota. Anthropogenic activities influence host-associated microbial communities through multiple interacting pathways, three of which are highlighted here: pollution, habitat transformation, and altered host contact networks. Pollution can drive the loss of co-evolved symbionts and increase selective pressures for the emergence and spread of antimicrobial resistance (AMR). Habitat transformation reshapes microbiota by changing habitat connectivity and microbial dispersal, as well as through physiological stress and host responses, often in a species-specific manner. Arrows indicate the direction of change in microbiota diversity across host species (red = decreased, green = increased, yellow = unchanged). Altered host contact networks result from human activities that reorganize which species interact, how often they come into contact, and which microbial source pools they encounter. These altered networks can create opportunities for human–wildlife microbial exchange, cross-species transmission, and microbial establishment in new host species or populations. Solid gray lines indicate existing wildlife interactions, broken gray lines indicate disrupted interactions, and purple bidirectional arrows indicate new or intensified anthropogenic connections. Together, these pathways can alter the composition, function, and evolutionary dynamics of microbiota, with implications for host health and disease risk. Created in BioRender. Moeller, A. (2026) https://BioRender.com/i9uzzmg.

In this Unsolved Mystery, we describe three major pathways through which humans are reshaping the microbiota of wild animals (Fig 1): pollution from human and domestic animal waste and other anthropogenic sources, including pesticide and antibiotic exposure; habitat transformation, including land-use change and climate-driven environmental shifts, which influence diet, host population structure, connectivity, and stress-mediated microbiota responses; and altered host contact networks, which create opportunities for microbes to cross species boundaries and establish in new hosts, including through human–wildlife contact, biological invasions, and feralization of domestic animals. This framework is not intended to encompass all possible human impacts on wildlife microbiota, nor to imply that these pathways act independently. Rather, it distinguishes the primary component of the host–microbe system initially perturbed (chemical/material environments, habitat structure, or host contact networks), while recognizing that these perturbations often interact to restructure wildlife-associated microbial communities across ecological scales. We review evidence of effects within each pathway and discuss how understanding human influences on wildlife microbiota—and the ecological and evolutionary dynamics of human-associated microbes in wild animal populations—will be important for safeguarding both human and animal health.

The mystery at the center of this article is not whether human activities affect wildlife microbiota, but why similar anthropogenic pressures produce different microbial responses across host species, ecological contexts, and timescales, and when those responses become biologically consequential. Human impacts may erode long-standing host–microbe associations, select for antimicrobial resistance (AMR), reshape microbial dispersal, alter host physiology, or create opportunities for cross-species microbial exchange. Yet it remains difficult to predict whether observed microbiota shifts are transient or persistent, harmful or adaptive, important or inconsequential for wildlife health, fitness, population dynamics, and disease risk. We use the framework above to examine this mystery and to discuss the research needed to distinguish mechanisms, measure consequences, and determine the conditions under which anthropogenic microbiota changes matter for wildlife hosts.

## How does pollution disrupt wildlife microbiota?

Some of the most ubiquitous effects that humans have on wildlife and their associated microbiota are mediated by environmental pollution [11]. In this section, we consider pollution by chemical and material contaminants, including heavy metals, pesticides, antibiotics, pharmaceutical effluent, and other toxicants, rather than biological inputs involving the direct introduction of live microbes. Some sources of pollution, such as wastewater or domestic animal waste, can also alter microbial source pools and facilitate microbial transmission; we treat those effects as interactions between pollution and altered host contact networks.

Exposure to chemical and material pollutants can reshape the structure, stability, and physiology of host-associated microbiota [12], which in turn influences how hosts process and respond to pollutants, including through microbial biotransformation and effects on host detoxification pathways. Thus, microbiota responses to pollutants may be beneficial, harmful, or context-dependent for hosts, but the consequences of these responses remain unclear for most wildlife species. Recent evidence shows that pollution can drive both loss of beneficial microbial taxa and adaptive changes via directional selection within wildlife microbiota, with potentially profound consequences for host health, resilience, and fitness (S1 Table). Together, these findings suggest that pollution not only reshapes microbiota composition but may also erode key symbiotic relationships that support host health.

### Loss of co-evolved symbionts

A wide range of environmental pollutants, including heavy metals, pesticides, and antibiotics, can alter the selective landscape experienced by microbiota within hosts, potentially driving the loss of microbiota constituents that have persisted for millions of years (Box 1). These changes may reflect declines in the relative abundance of sensitive taxa or shifts in community evenness, but they may also be underlain by local extinction of symbionts from host individuals or populations, a hypothesis that warrants further study. Erosion of co-evolved symbioses critical for digestion, immunity, or development may weaken host resilience to environmental stress and reduce fitness. Reductions in microbial diversity within individual hosts, for example, can decrease colonization resistance to pathogen invasion [13,14]. The risks of microbiota erosion may be particularly acute in insects, where symbionts are often obligate, less diverse, and provide direct protection against pathogens and parasites [15,16]. In bees, for example, microbiota disruption is implicated in colony collapse disorder [17], a phenomenon with severe consequences for agriculture. Although pollutant-induced losses of microbiota are often deleterious for hosts, in some cases, they may open ecological niche space for taxa that aid pollutant detoxification (Box 1). For instance, parasitic helminths that interact with the microbiota in host guts significantly reduce the concentration of environmental contaminants [18,19]. A major direction for future work will therefore be field- and laboratory-based tests of the phenotypic consequences of pollution-mediated reductions in microbiota diversity.

**Box 1. Pollution-induced losses of symbiont diversity from wildlife microbiota**

**Heavy metals and radionuclides**

Mining, nuclear accidents, and related industrial activities are major sources of environmental metal and radionuclide contamination that can alter wildlife microbiota. Tree sparrow nestlings show decreased gut microbiota diversity (alpha diversity) and increased inter-individual variation (beta diversity) in metal-polluted areas, alongside increased relative abundances of functional pathways associated with metal toxicity resistance [20]. Earthworms inhabiting arsenic-contaminated soils lose several bacterial taxa [21], and exposure to environmental concentrations of cadmium also alters earthworm gut microbiota, increasing the relative abundance of heavy-metal-resistant bacteria [22]. Similarly, wild *Apodemus* mice exposed to radionuclide contamination experience shifts in community composition in both general and host-specific ways, despite unchanged alpha diversity [23], suggesting pollutant-mediated loss and replacement of endogenous microbial taxa. Conversely, free-living bank voles (*Myodes glareolus*) exhibit higher gut microbiota diversity as well as altered community composition in metal-contaminated environments; this observation could suggest greater resistance to environmental perturbations and pathogen invasion, or reflect reduced stability or increased richness of opportunistic pathogens [24].

**Pesticides**

Numerous laboratory studies demonstrate that pesticide exposure reshapes gut microbiota composition, increases gut permeability and low-grade inflammation, and can even affect host behavior through microbially derived metabolites, such as short-chain fatty acids, which influence appetite [25–29]. In wildlife, pesticide exposure risk is not confined to agricultural ‘hot-spots’: free-living small mammals show widespread contamination by both legacy and currently used pesticides, often at high concentrations [30]. Across host taxa, pesticide exposure perturbs microbiota [31], driving the loss of microbial diversity or turnover in microbiota composition. For instance, experimental exposure to the herbicide glyphosate at environmental concentrations drives decreases in the abundance of dominant gut bacteria in bees [32]. Pesticide-induced losses of microbiota may contribute to deleterious effects on host digestion, immunity, and development. For example, the widely used organophosphate chlorpyrifos promotes obesity and insulin resistance in mice via microbiota interactions [33]. Yet, effects may not be uniformly negative: in some contexts, altered microbiota may enhance host resilience by contributing to pesticide detoxification [34]. Moreover, the neonicotinoid imidacloprid reduces bee survival without altering microbiota [35], indicating that negative health impacts of pesticides can occur independently of microbial disruption.

**Antibiotics**

Antibiotics constitute another class of widespread pollutants that can erode co-evolved symbioses. These pollutants enter ecosystems through industrial and pharmaceutical effluent, wastewater discharge, and agricultural runoff, and are inefficiently removed by standard treatment methods, often becoming concentrated downstream [36]. Laboratory studies show that exposure of the microbiota to antibiotics drives sharp declines in taxonomic diversity as well as persistent shifts in metabolic potential and colonization resistance [37–40]. Laboratory zebrafish exposed to environmentally realistic levels of sulfamethoxazole and oxytetracycline display altered gut communities, increased inflammation, reduced immune enzyme activity, and impaired resistance to the pathogen *Aeromonas hydrophila* [41]. Tadpoles in an artificial freshwater system with antibiotic pollution experience turnover in microbiota composition, resulting in disrupted metabolism and reduced fitness [42]. Parallel findings are emerging from studies of wildlife: human disturbance alters gut microbiota structure and function in golden snub-nosed monkeys (*Rhinopithecus roxellana*), with disproportionate effects on Firmicutes (often associated with fiber metabolism) and little discernible impact on Proteobacteria, which can increase under conditions of host metabolic stress [43]. Similarly, wild goitered gazelles (*Gazella subgutturosa*) inhabiting antibiotic-contaminated areas in the Qaidam Basin exhibit reduced microbial diversity and significant compositional shifts, which may simultaneously buffer metabolic stress and heighten inflammatory risks [44].

### Selection for antimicrobial resistance

In addition to disrupting beneficial symbioses, antibiotic pollutants can directly select for AMR, while pesticides and heavy metals can promote AMR through co-selection mechanisms in environmental and host-associated microbial communities. Wildlife in polluted landscapes, especially near wastewater effluent, livestock operations, or urban centers, often harbor bacteria carrying clinically relevant antimicrobial resistance genes (ARGs), effectively transforming wildlife into reservoirs or amplifiers of AMR [45–47]. In these settings, pollution can influence AMR through at least two non-exclusive processes: chemical selection, in which antibiotics, heavy metals, pesticides, or other contaminants favor resistant bacteria or resistance genes; and microbial input, in which wastewater, livestock waste, or other sources introduce resistant bacteria and mobile genetic elements into environmental reservoirs encountered by wildlife. Although environmental concentrations of antibiotic pollutants are lower than those used in clinical or agricultural settings, sub-inhibitory levels can in some cases select for resistance more rapidly than full dosages [48]. Non-antibiotic pollutants can also promote AMR through co-selection, including: co-resistance, in which genes conferring resistance to antibiotics and metals or biocides are physically linked on plasmids, transposons, integrons, or other mobile genetic elements; cross-resistance, in which a single mechanism, such as a multidrug efflux pump, or reduced membrane permeability, confers tolerance to multiple compounds; and co-regulation, in which exposure to one agent triggers regulatory responses that increase tolerance to others [49]. In one in vitro study, *Escherichia coli* evolved resistance up to 100,000 times faster when exposed to an antibiotic–herbicide combination compared to the antibiotic alone [50]. These co-selection processes may be amplified when mobile genetic elements carrying ARGs spread among bacterial taxa through horizontal gene transfer (HGT) [51]. Together, these processes position polluted landscapes to function as interfaces where anthropogenic selection acts across wildlife microbiota and environmental reservoirs to shape the emergence, persistence, and spread of clinically relevant resistance.

Wildlife are increasingly recognized as potential reservoirs and disseminators of clinically relevant resistance [43,52–56]. The prevalence of ARGs in wildlife microbiota is strongly associated with human antibiotic use and the resulting environmental contamination, with higher frequencies typically observed near human development [57]. Viruses, including those that infect bacteria (bacteriophages), are a key reservoir of AMR in the environment [58], and their diversity and abundance are directly driven by the selection pressure of their environment (or host) [59,60]. This suggests that wildlife microbiota not only reflect anthropogenic pressures but may also serve as early indicators of ecological disturbance and human health risk. Temporal studies demonstrate the value of wildlife as sentinels: shotgun metagenomic analysis of dental calculus from wild Swedish brown bears spanning the pre- and post-antibiotic eras showed that AMR levels tracked national antibiotic use over decades, with a decline following control policies [61]. Such evidence underscores the idea that interventions can meaningfully reduce the prevalence of resistance. However, recent findings complicate this narrative. For example, >30% of *E. coli* isolates from wild birds and mammals were resistant to colistin (a last-resort antibiotic), yet the prevalence of resistance did not reflect the level of human impact at sampling sites [62]. Indeed, clinically relevant ARGs have been detected in animals from remote regions, including the Arctic [63] and the Gobi Desert [64]. Such results could reflect several non-exclusive processes: natural background resistance maintained by microbial competition, widespread anthropogenic contamination that masks local gradients, or long-distance dispersal of resistant bacteria, ARGs, and selective agents via wind, water, or animal movement. Migratory birds, in particular, have been highlighted as major vectors for the dissemination of resistance across large geographic scales [65], with urban proximity linked to higher AMR incidence in wild bird populations [66]. A major unresolved challenge is therefore to distinguish natural resistome variation from pollutant-driven proliferation of ARGs in wildlife microbiota. Addressing this challenge will require replicated sampling across anthropogenic exposure gradients, paired measurements of pollutants and microbial source pools, and approaches that distinguish background resistance from ARGs bearing signatures of anthropogenic influence, such as clinical relevance, mobility, or recent acquisition. Strain-resolved metagenomics, reconstruction of ARG genomic context, mobile-element tracking, absolute-abundance measurements, and phylogenetic comparisons with human-, livestock-, and environment-associated bacteria would help identify when ARGs in wildlife reflect natural microbial ecology versus recent anthropogenic selection or transmission.

**Fig 2:**
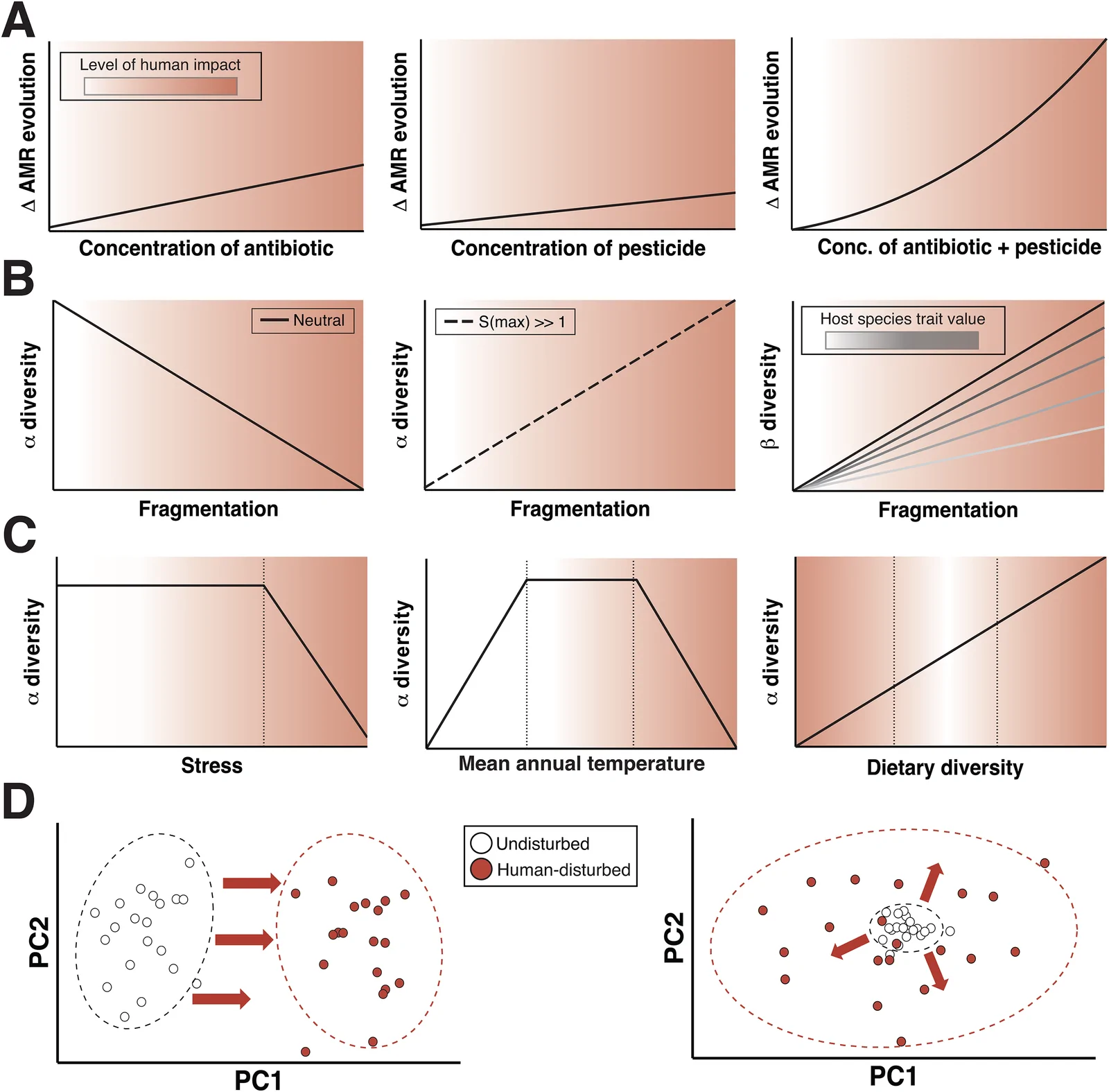
Conceptual predictions of anthropogenic impacts on microbiota diversity and community dynamics. Each panel isolates a focal process or hypothesis rather than representing a single composite gradient of human impact; in real systems, these processes often co-occur and require factorial, longitudinal, space-for-time, or gradient-based study designs to disentangle. **A.** Left: The rate of antimicrobial resistance (AMR) evolution increases with the level of antibiotic contamination in the environment, though sub-inhibitory levels of antibiotics may select for resistance faster than higher concentrations. Middle: Pesticides can co-select for AMR, though these relationships are not necessarily linear, and the presence of helminths may dampen or amplify the strength of this relationship. Right: Microbial communities evolve AMR at a faster rate (exponentially) when in the presence of both antibiotic and pesticide than either chemical alone. **B.** Left: Alpha (α) diversity decreases with habitat fragmentation under neutral theory. Middle: Alpha diversity increases with fragmentation when the maximum selection coefficient of a given species is much greater than one. Right: Habitat fragmentation increases population-level gut microbiota beta (β) diversity, but certain host species’ traits (e.g., degree of sociality/territoriality, home-range size, body size, dispersal ability) impact the strength of this relationship. **C.** Left: Stress associated with anthropogenic pressures could cause a loss of alpha diversity (e.g., gastrointestinal tract infection, impacts of starvation). Certain types of stress could lead to an increase in alpha diversity (e.g.*,* traumatic wound infection, lesser control by the host’s immune system). Nonlinear or threshold-like responses are shown where they have been proposed or empirically observed, but similar nonlinearities may occur for other anthropogenic pressures. Middle: In ectotherms, temperature has a major role in alpha diversity, with an optimal temperature range that supports the highest diversity. The plot shows a symmetrical relationship, but the resilience of microbiota diversity may differ between high and low temperature extremes. Right: Alpha diversity is predicted to increase with dietary diversity. Human impacts may either increase or decrease the dietary diversity accessible to wildlife, depending on the type of disturbance and the dietary flexibility of the host species. Microbiota diversity may also saturate with increasing dietary diversity, and these saturation effects may vary among dietary categories (e.g.*,* carnivores vs. omnivores). Furthermore, dietary diversity may vary seasonally. For example, wild mice experience pronounced seasonal dietary variation associated with microbiota diversity [68]. **D.** Hypothetical principal coordinate plots show two contrasting predictions for how beta diversity changes in response to human disturbance. Left: The individuals might experience parallel shifts in community composition, indicative of directional selection. Right: Human disturbance might result in stochastic dysbiosis among individuals, increasing beta diversity, reflecting the Anna Karenina Principle. These relationships are intended to be conceptual and non-exhaustive; in many cases, anthropogenic pressures may produce context-dependent or even opposing effects (e.g., reductions in AMR under certain pollutant regimes or decreased beta diversity under habitat homogenization), depending on ecological context, host traits, and local environmental conditions. PC1, principal component 1; PC2, principal component 2.

By favoring resistant bacteria and resistance-bearing mobile genetic elements, pollution exposure may increase the emergence, persistence, and spread of clinically relevant AMR in wildlife-associated and environmental microbial communities. For antibiotic pollutants, measured environmental concentrations can be sufficient to select for resistance in clinically relevant bacteria [67]. However, major knowledge gaps remain regarding how resistance enters, persists, and evolves in wildlife microbiota. Most research emphasizes screening for ARGs, with far less attention to the mechanisms by which resistance enters and persists in wildlife microbiota, or to the downstream ecological and evolutionary consequences. Even less is known about the effects of complex pollutant mixtures, which represent the most realistic exposure scenarios. Mixture effects can be additive, synergistic, or antagonistic, depending on the pollutants and bacterial taxa involved [49]. Conceptually, increasing concentrations of antibiotics and pesticides are expected to increase rates of AMR evolution, with potential synergistic effects when both are present (Fig 2A). Most studies to date rely on indirect comparisons of 16S rRNA profiles and ARG distributions across environmental samples [69,70], controlled in vitro assays [50]*,* or experiments using simplified microbial consortia and limited chemical combinations [71,72]. Recent advances in strain-resolved, low-cost metagenomics [8,73] and the use of germ-free animal models [74,75] now make it possible to trace how complex pollutant mixtures shape resistance at the strain level in vivo. Because causal links between AMR, pollutant exposure, and wildlife health are often ethically and logistically constrained, future work should combine observational, longitudinal, and experimental approaches to examine how pollutants interact with other anthropogenic pressures, including habitat transformation and cross-species microbiota transmission, to shape host outcomes.

## How does habitat transformation reshape wildlife microbiota?

In addition to introducing pervasive environmental pollutants, humans have extensively modified the landscapes of terrestrial and marine ecosystems: by 2018, over 77% of land (excluding Antarctica) and 87% of the ocean had been directly impacted by human activity [76,77]. Such habitat transformation reshapes the structure and connectivity of animal populations, with downstream effects on animal-associated microbial diversity and function, and potential consequences for host health. Numerous studies show that human encroachment into wild spaces affects the gut microbiota of nearby wild species [45–47,78–86]. In this section, we focus on urbanization as a major form of anthropogenic habitat transformation, while recognizing that urbanization can also introduce pollutants and increase contact among humans, domestic animals, and wildlife. We emphasize how urbanization changes landscape structure, resource availability, thermal environments, host physiology and stress responses, microbial dispersal within wildlife populations, and species-specific responses. We return to cross-species microbial transmission in the section on altered host contact networks.

### Habitat connectivity and microbial dispersal

Urban expansion is an accelerating global driver of habitat loss and biodiversity decline. Urban areas are expanding faster than any other land cover type [87], with a predicted 200% increase in global urban land cover by 2030 compared with the year 2000 [88]. Habitat degradation and urbanization have often been associated with reductions in microbiota diversity within wildlife individuals (i.e., alpha diversity) [82,89,90]. Because microbiota responses vary across host species and environmental contexts, broader syntheses have yet to identify consistent general patterns. For example, urbanization does not uniformly reduce microbiota alpha diversity: some urban-dwelling wildlife exhibit higher within-host diversity [91,92], while others show no clear correlation with human impact [86,93–95].

The conflicting effects of urbanization and habitat fragmentation on microbiota reported in prior literature can be reconciled through the lens of metacommunity theory. Habitat fragmentation may decrease dispersal and limit microbiota exchange among conspecific hosts [96]. Under a neutral model of microbiota assembly, in which fitness differences among microbial species are small or nonexistent, such reductions in dispersal are expected to reduce alpha diversity, as limited immigration constrains local community richness. However, empirical studies do not always conform to this prediction: geographic barriers can increase divergence in microbiota composition among host populations, even when effects on alpha diversity are variable or context-dependent. When fitness differences among microbial species are large, reduced dispersal can instead increase within-host alpha diversity [97] (Fig 2B). Under high connectivity, competitively dominant taxa may spread throughout a metacommunity (i.e., network of hosts), thereby suppressing diversity that could otherwise be maintained under more fragmented conditions.

More broadly, fragmentation can reshape the selective landscape experienced by microbial communities by altering habitat connectivity, local environmental conditions, and host population structure, thereby influencing which taxa persist and spread across the metacommunity [96]. Host traits such as home range, diet, sociality, and adaptability can further shape microbiota responses to habitat fragmentation [98], suggesting that some microbial species are more vulnerable to habitat disruption than others. Host-species-specific assessments that consider how host ecology, dispersal limitation, and stochastic processes interact to shape microbiota responses, including understudied components such as eukaryotes and phages, will be critical for reconciling the variable effects of urbanization on microbiota diversity.

### Physiological and ecological drivers of microbiota assembly

In addition to distorting microbiota dispersal dynamics, urbanization exerts multifaceted selective pressures on wildlife microbiota. These include dietary shifts, abiotic stressors such as temperature, and physiological stress responses, all of which can reshape microbial community structure and function.

Urbanization can alter wildlife dietary composition via modified foraging landscapes, which in turn can influence microbial community structure independent of fragmentation per se [83,99–103]. Past work has reported positive associations between dietary diversity and microbiota diversity, such that reduced dietary diversity in urban environments is often associated with lower alpha diversity (Fig 2C), although these relationships are often correlative and may not reflect direct causal effects. Thus, the negative effect of urban-associated diets on alpha diversity may not be universal and likely reflects context-dependent interactions among diet, habitat, and host ecology. For example, urban generalists, such as rats or raccoons, may have more diverse diets in urban settings relative to undisturbed settings (Fig 2C).

Climate change and urbanization can also alter the thermal environments experienced by wildlife, which in turn can influence wildlife microbiota [104]. Experimental work in ectotherms, including lizards and amphibians, has shown that microbial communities respond to temperature in ways that shape host physiology and, in some cases, thermal tolerance [105–107]. Interestingly, the effects of warming on microbiota composition can interact with the effects of alterations to habitat connectivity: a recent experiment in lizards showed that increasing connectivity between subpopulations could buffer against the effects of warming on the microbiota [108].

Microbiota alterations associated with urbanization may also arise through host stress responses to habitat disturbance [109]. For example, comparisons of forest- and urban-dwelling eastern gray squirrels (*Sciurus carolinensis*) indicate that stress-related physiological changes mediated by the hypothalamic-pituitary-adrenal axis correspond with shifts in gut microbiota [110]. Thermal stress may act through similar physiological pathways: experiments in amphibians demonstrate that increased temperatures can activate stress axes, alter microbiota composition, and modulate immune function [111]. Together, these findings highlight stress-mediated effects as an indirect pathway through which anthropogenic environments shape host-associated microbial communities.

Like pollution, dietary and temperature-related stressors, which are often associated with urban environments, can exert directional selection on microbiota composition, although separating these effects from urbanization per se remains challenging without a baseline understanding of how wildlife microbiota respond to diet and temperature in non-urban systems. Directional changes in microbiota composition could be deleterious for hosts, but they may also mediate host adaptation or acclimatization to the novel environments, such as those imposed by urban heat islands [112]. Selection can also be disruptive, leading to increased heterogeneity of microbiota composition among hosts in urban environments relative to undisturbed environments (Fig 2D) [113]. These dynamics reflect the Anna Karenina Principle, whereby disturbed hosts exhibit more variable and less predictable microbiota than undisturbed hosts, indicating a shift toward stochastic, host-specific trajectories [114,115] (Fig 2D).

### Species-specific responses to habitat transformation

Microbiota responses to anthropogenic habitat transformation vary systematically across wildlife species, reflecting differences in host ecology and life-history traits. Key traits shaping these responses include home-range size, sociality, dietary breadth, and intrinsic stress tolerance. For example, distantly related sympatric generalist species showed divergent microbiota responses to habitat fragmentation [98]. These traits may also vary within host species: in eastern gray squirrels, urbanization impacts differed by coat color phenotype, which is often pleiotropically linked to numerous physiological pathways [116]. Most studies to date have focused on individual species, often using disparate sequencing and analytical approaches (S2 Table), limiting cross-species comparisons. Comparative studies across host species with differing lifestyles (e.g.*,* solitary versus social, home-range size) and diet (e.g., carnivore, herbivore, omnivore) will be essential for identifying general principles governing microbiota responses to habitat transformation. Another axis of variation is species’ tolerance to urban environments. Species can be broadly categorized based on their response to urbanization: urban dwellers, which have specialized to the extent that they live at the expense of humans; urban adapters, which can adapt to and tolerate urban living; and urban avoiders, which are unable to survive in urban areas [117]. Some species may benefit from anthropogenic environments, which provide access to human food sources and relief from competition and predation. For instance, urban-dwelling small mammals harbor reduced taxonomic diversity but increased functional diversity in their microbial communities (indicating adaptation to urban environments despite dysbiosis) compared with urban adapters, which display relatively stable composition and higher functional redundancy (indicating a potential to adapt to urban living), whereas urban avoiders display uniformly negative responses in taxonomic and functional diversity [118]. Together, these patterns highlight that host ecology modulates how anthropogenic pressures alter microbiota.

The variation in effects of urbanization on microbiota across wildlife species implies that strategies to mitigate these effects [119–121] will need to consider host-species-specific responses. For example, in rodents, exposure to green spaces in otherwise urban environments was enough to provide resilience in the microbiota [122], but this mitigation strategy may not suffice for larger species or those with more expansive territory requirements. Moreover, incorporating quantitative models of habitat fragmentation will help standardize assessments and move beyond categorical ‘undisturbed versus disturbed’ classifications [123]. Because microbiota can respond rapidly to environmental change, they may serve as sensitive indicators of habitat fragmentation and reduced connectivity, with shifts potentially detectable long before corresponding changes in host genomic structure [96]. These insights also open opportunities for microbiota-informed interventions, such as restoring or maintaining exposure to environmentally derived microbial communities or designing habitats that support beneficial microbial exchange among hosts.

## How do altered host contact networks facilitate microbial exchange and invasion?

In addition to chemical pollutants and habitat-mediated changes in selection, human activities can reshape wildlife microbiota by reorganizing the contact networks through which microbes disperse, creating new opportunities for transmission across host species that did not interact prior to disturbance. Such reorganization can increase direct or indirect contact among humans, domestic animals, invasive and feral species, and wildlife, including at human–wildland interfaces and during biological invasion or feralization. Microbial invasions are therefore not independent of pollution or habitat transformation; they often arise when these anthropogenic pressures change microbial source pools, host contact rates, or opportunities for colonization. In this section, we focus on cases in which altered host contact networks allow microbes to cross species boundaries, establish in new hosts, encounter new selective pressures, and evolve.

### Microbiota exchange between humans and wildlife

Many devastating human diseases such as influenza, smallpox, HIV, Ebola, and the bubonic plague have zoonotic origins [124]. Accordingly, much research has focused on the spillover of pathogens from wild animals into human populations. This one-way framing has shaped interventive actions, including wildlife displacement or culling for disease prevention. However, pathogen flow is bidirectional: recent analyses suggest that humans transmit roughly twice as many viruses to animals as we acquire from them [125]. Nevertheless, comparatively little attention has been paid to the consequences of human-to-wildlife pathogen transfer [126]. Calls for a ‘One Health’ perspective underscore that the health of humans, animals, and ecosystems is deeply interconnected and highlight the need to understand these bidirectional flows in the context of global change pressures [127].

**Fig 3:**
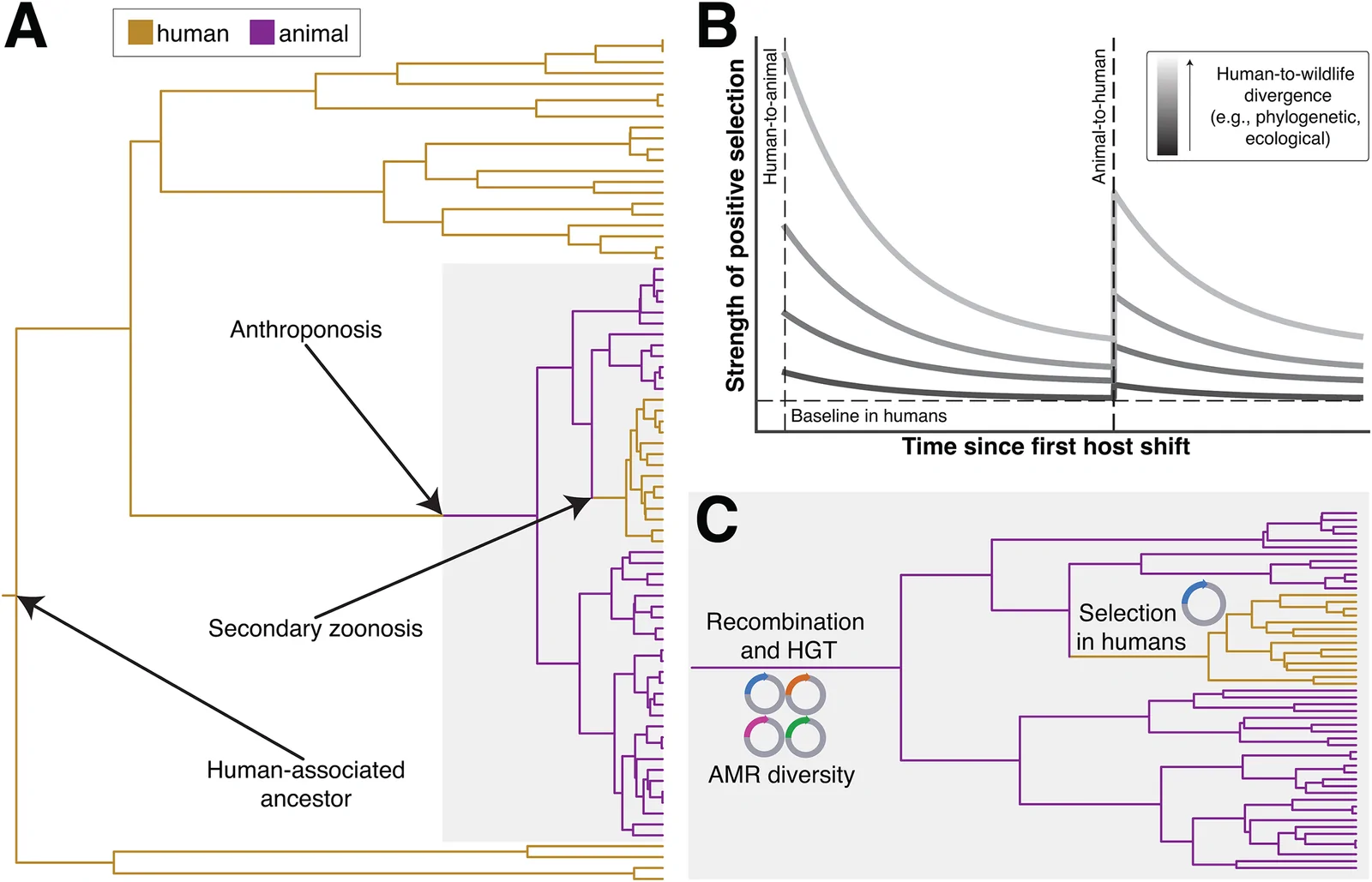
Phylogenetic signatures of secondary spillover. **A.** Phylogenetic tree shows relatedness patterns indicative of human-to-animal-to-human transmission of a human-associated microbe, wherein initial spillback (anthroponosis) is followed by a secondary zoonotic spillover event, after which the microbe is again detected in humans. **B.** When a microbe successfully establishes in a new host, it faces novel selective pressures, the strength of which is expected to increase with greater human-to-wildlife phylogenetic or ecological divergence. Although spillover between closely related hosts (e.g., primates) may be more likely to result in successful transmission, successful colonization in more divergent hosts is expected to involve stronger selective pressures. **C.** After anthroponotic spillover, selective pressures in wildlife may favor proliferation, recombination, and horizontal gene transfer (HGT) of microbial traits that facilitate colonization (and, potentially, pathogenesis) in the new host, including, in some cases, antimicrobial resistance (AMR) or virulence-associated factors. After secondary spillover, a subset of these traits may be retained or further selected for in humans, for example, under antibiotic exposure.

Clarifying the multiple modes of interspecific transmission is a prerequisite for disentangling how human-derived microbes move among host species and the risks they may pose. Microbiota exchange between humans and wildlife can occur through direct contact or indirectly via airborne, waterborne, foodborne, or vector-borne routes. In this context, spillover (zoonosis) refers to animal-to-human transmission, spillback (anthroponosis) to human-to-animal transfer, and secondary spillover to cases in which a pathogen re-enters human populations after establishing in a non-human host, a process that can be detected from characteristic phylogenetic relatedness patterns of human- and animal-associated microbial lineages (Fig 3A).

When microbes cross species boundaries, they encounter unfamiliar immune landscapes and ecological conditions, often shaped by anthropogenic stressors. These transitions can accelerate microbial adaptation, driving the evolution of traits such as heightened virulence, AMR, and host-range expansion. Thus, interspecific transmission represents both a conservation concern and a driver of microbial evolution. Anthropogenic effects, including widespread urbanization, are accelerating human-to-wildlife microbial transmission, which can accompany and interact with anthropogenic microbiota disruptions within wildlife species.

Emerging evidence suggests that human-associated gut microbes can colonize wildlife in disturbed settings, such as urban or captive environments, contributing to the ‘humanization’ of wildlife microbiota [81], although the relative roles of direct transmission versus parallel environmental selection remain unresolved. Meta-analyses of hundreds of gut microbiota profiles have shown that living in captivity (e.g., in zoos) drives directional changes in mammalian microbiota composition toward a human-like state [81,128]. However, these studies did not explicitly track microbial strain origins, leaving unresolved how much observed shifts toward human-associated community states reflect direct transmission versus shared diets, antibiotic exposure, or other environmental factors. Moreover, whether human-associated taxa detected in wildlife represent transient exposure, stable colonization, or functionally important members of the microbiota remains largely unknown. A recent study reported similar shifts toward human-associated microbiota in urban environments, where synanthropic species, including coyotes, lizards, and sparrows, harbor gut microbes that appear to be derived from humans [80]. Such mismatches between hosts and microbiota may have important consequences for wildlife health and could facilitate downstream evolutionary dynamics, including secondary spillover following sustained establishment in novel hosts.

Secondary spillover, though difficult to detect and therefore likely underreported, has been documented in several systems [126]. During the SARS-CoV-2 pandemic, white-tailed deer (*Odocoileus virginianus*) were found to harbor human-derived SARS-CoV-2 lineages that evolved up to three times faster in deer than in humans [129], with at least one documented deer-to-human transmission [130]. Human activities that generate opportunities for human-to-animal-to-human transmission include animal husbandry and wildlife trafficking [131–133]. Additional examples include pathogens transmitted from humans to cattle, deer, and other species, with evidence of reintroduction into human populations [134–138]. These cases illustrate that spillback is not an ecological dead end; rather, secondary spillover can create feedback loops that amplify disease risks for both wildlife and people.

While most prior work on secondary spillover has focused on viruses, there is a growing need to understand these dynamics in bacterial pathogens, particularly given recent evidence of human-to-wildlife microbiota transmission in captive and urban settings. Recent work indicates that bacterial transmission among humans and wildlife may be underappreciated by surveillance programs [126]. Once human-derived bacteria colonize wildlife hosts, their persistence, ecological impacts, and evolutionary trajectories become critical considerations. These microbes may establish stable populations or remain transient invaders, alter existing microbiota and host–microbe interactions, and experience novel selective pressures associated with new immune environments and ecological niches (Fig 3B). In doing so, they may acquire or amplify traits such as AMR or expand host range (Fig 3C), while also disrupting resident microbiota through competitive exclusion or HGT, with cascading effects on host nutrition, immunity, and disease susceptibility. Understanding these within-host processes is crucial for determining how interspecific transmission reshapes host-associated microbial ecology and pathogen evolution. Phylogenetic analyses of genome-resolved metagenomic datasets provide a powerful framework for identifying cases of secondary spillover by tracing the evolutionary trajectories of human-derived microbes across host-species boundaries, particularly when integrated with ecological and epidemiological data. Integrating these approaches with ecological monitoring will help clarify when transient colonization becomes stable establishment and when such shifts amplify the risk of novel disease emergence.

### Microbial source pools associated with invasive and feral hosts

Beyond transmission events between humans and wildlife, human-mediated species redistribution (including invasive species and feralization of domestic species) can further restructure microbial exchange networks at broader spatial and evolutionary scales. Human activity has not only reshaped habitats and ecosystems, but has also accelerated the global redistribution of species, creating unprecedented opportunities for biological invasions and altering the landscape of microbial transmission. More than one-third of terrestrial land area now contains established populations of non-native vertebrates that were introduced either intentionally (e.g., livestock, pets, biocontrol) or inadvertently through trade, transport, and habitat disturbance [139]. Invasive species are a leading driver of biodiversity loss, ecosystem degradation, and pathogen emergence, and are estimated to cost the global economy over $400 billion USD annually [140]. Yet, invasion biology has only recently begun to investigate the consequences for wildlife microbiota. Similarly, there is a need to consider the influence of microbiota on the success of biological invasions, given that microbiota can influence ecological performance, niche expansion, and rapid adaptation to novel environments [141].

Some of the most significant invasive species are ex-domesticates; in a process known as ‘feralization’, domesticated animals can revert to self-sustaining wild populations. Feral taxa, such as pigs (*Sus scrofa*), goats (*Capra hircus*), horses (*Equus caballus*), cats (*Felis catus*), and dogs (*Canis familiaris*), are among the world’s most ecologically destructive invaders. Feralization differs from classical invasion in that the starting population has already undergone strong artificial selection for traits such as rapid growth, reduced fear response, and/or dietary flexibility. Moreover, domesticated species often exhibit lower genomic diversity than their wild counterparts and may therefore lack the genomic substrate of standing variation on which selection can act during the process of feralization. This domestic legacy may extend to the microbiota: domesticated mammals typically harbor less diverse, carbohydrate-adapted microbiota compared with their wild relatives [142], raising the question of whether feralization involves microbiota ‘re-assembly’ and whether failure to reacquire native microbes constrains fitness or adaptive capacity [143]. Importantly, while such constraints could limit the success of invasive feral populations, they may conversely represent a conservation concern in rewilding contexts, where species are reintroduced to their native ranges. Thus, understanding microbiota dynamics in feral populations may help guide microbiota-informed conservation strategies. Conversely, microbiota diversity may be more robust to domestication, such that phenotypic changes during feralization may be mediated by the microbiota rather than by host genomic evolution. In line with this possibility, recent experimental work in mice shows that behavioral phenotypes can adapt through selection on the microbiota alone even in the absence of host genomic variation [144].

Recent theory distinguishes endo-feralization (adaptation relying primarily on standing genetic variation within the domestic lineage) from exo-feralization, in which feral populations acquire genetic material from closely related wild taxa via hybridization. Although originally framed in terms of host genomes, this endo/exo distinction also applies to gut microbial constituents. In endo-feralization, microbiota shifts may arise from selection on existing strain diversity, or from exposure to novel environmental reservoirs (e.g., soil, water, prey, carrion). In exo-feralization, hybridization with close relatives in the wild may result in the establishment of chimeric microbial communities composed of constituents from both host parent species [145]. Supporting this idea, recent comparative genomic studies have suggested extensive microbial transmission between wild and domesticated animals [146]. Even in the absence of hybridization, exo-feralization of microbiota may proceed via horizontal acquisition of microbial lineages from wild animals through ecological interactions and shared environmental exposures that facilitate exchange between hosts. A handful of studies have addressed this process in feral animals: feral boar carry bacteria associated with both domestic pigs and wild boars, as well as some exclusive taxa, suggesting recent re-adaptation to the wild [147]; and feral cat microbiota show significantly greater functional capacity than those of domestic cats [148]. These functional shifts were also associated with heightened aggression and elusive behavior in feral cats, suggesting that microbiota changes may contribute to physiological and behavioral traits advantageous for a feral lifestyle. However, these patterns may also reflect host genetic differences, including hybridization with wild relatives, highlighting the need for future work to quantify the relative contributions of host genetic variation and microbiota change to adaptation or acclimation during feralization. For example, studies pairing host genomic analyses with metagenomic profiling could help identify host genetic and microbial factors associated with adaptive variation in feral traits.

The microbiota may also contribute to invasion success in non-domesticated species. Invasive populations frequently encounter new diets, competitors, pathogens, and abiotic stressors. Microbiota-mediated phenotypic plasticity, whereby changes in microbiota composition or function alter host metabolic capacity, detoxification, or immune responses, may explain how some invaders achieve rapid ecological release despite these challenges [149]. In addition, the acquisition of novel microbial taxa may further facilitate invasion success. Recent theoretical work suggests that microbiota transfer from native to invasive species may facilitate invasion success [150]. Furthermore, comparing parasite burdens in invasive species to parasite burdens in native ranges suggested that parasite diversity declines precipitously in species after successful invasion, whereas the acquisition of local pathogens can require decades [151]. Such invasion-associated bottlenecks may also reduce the microbiota diversity of successful invaders during the early establishment period. These findings raise the possibility that symbionts, not just host genetics, are relevant in biological invasions—and that microbiota may broaden the realized niche of invasive species, alter competitive hierarchies with native fauna, and even accelerate ecosystem-level change. As with feralization, future work will need to disentangle the relative contributions of microbiota-mediated phenotypic plasticity and heritable host adaptation to invasion success in non-domesticated species.

Feral and invasive species also contribute to microbial exchange between humans, livestock, and wildlife. Feral swine carry numerous zoonotic pathogens capable of infecting humans, including brucellosis, bovine tuberculosis, leptospirosis, enteric pathogens such as *Salmonella* spp. and Shiga toxin-producing *E. coli*, and hepatitis E, all of which can be transmitted to humans by direct contact or by consumption of feral swine meat [152]. Feral cats in coastal California host a high prevalence and diversity of *Toxoplasma gondii*, which can be transmitted to people and other wildlife such as sea otters [153]. These examples illustrate that invasive species not only restructure local food webs, but also change the area’s metacommunity of microbes, creating new opportunities for interspecific transmission.

## What will it take to understand when human-induced changes to wildlife microbiota matter?

Building on the pathways reviewed above, we see three major future directions for understanding when human-induced changes to wildlife microbiota matter. First, there remains a need to understand how microbiota shifts influence host outcomes, including physiology, immunity, behavior, reproduction, survival, and other fitness-relevant traits. Second, future work will need to disentangle how interacting anthropogenic pressures jointly shape wildlife microbiota, because processes such as urbanization can simultaneously transform habitats, introduce pollutants, and increase opportunities for cross-species microbial exchange. Third, it will be important to broaden studies beyond bacterial taxonomic profiles to include strain- and function-resolved metagenomics, metabolomics, viromics, mycobiome profiling, and other understudied members of host-associated microbial communities. Across all three directions, progress will require study designs and methodological approaches that move beyond documenting microbiota shifts and towards identifying mechanisms and consequences. Because causal experiments in wildlife are often constrained by ethical, logistical, and temporal challenges, this effort will require combining long-term field studies, time-series and space-for-time designs, minimally invasive sampling, comparative approaches, and targeted experimental systems, where appropriate (Box 2).

**Box 2. Study designs and tools for understanding when human-induced changes to wildlife microbiota matter**

**1. Link microbiota shifts to host outcomes**

Pair microbiota profiling with measurements of host physiology, immune function, behavior, reproduction, survival, or other fitness-relevant traits.

**2. Leverage long-term and comparative field designs**

Use repeated sampling across anthropogenic gradients, space-for-time designs, before–after disturbance studies, habitat restoration or remediation studies, measurements of environmental microbial source pools, and long-term ecological monitoring initiatives such as the US National Science Foundation’s National Ecological Observatory Network to strengthen causal inference. These approaches can help test whether microbiota changes reflect altered selection, microbial dispersal, cross-species transmission, or their interaction.

**3. Move beyond bacterial taxonomic profiles**

Incorporate shotgun metagenomics, metabolomics, viromics, mycobiome profiling, and parasite or microeukaryote surveys to characterize functional potential, realized metabolic outputs, and understudied members of wildlife microbiota.

**4. Test mechanisms experimentally when feasible**

Use ethically appropriate mesocosms, controlled exposure studies, captive or rehabilitation settings, and gnotobiotic models to test how anthropogenic pressures alter colonization resistance, microbial persistence, and host responses.

### What effects do anthropogenic microbiota alterations have on wildlife phenotypes?

A central unresolved question is how human-induced changes in wildlife microbiota influence host phenotypes. Progress has been limited by the predominance of studies using 16S rRNA gene amplicon sequencing, which provides limited information about strain-level variation and functional potential, together with observational and single-time point sampling designs that make it difficult to infer causality or resolve temporal dynamics (S1 and S2 Tables). As a result, the phenotypic consequences of microbiota disruption—whether deleterious, neutral, or adaptive—remain poorly understood.

Evidence from model systems suggests that colonization by non-native gut microbial communities can disrupt host growth and immune development [75,154], raising the possibility that similar processes occur in wildlife. One compelling hypothesis is that gastrointestinal dysfunction observed in many animal species under human influence (e.g., in captive breeding programs) [120,155,156] may be attributable to human-induced microbiota alterations, including evolutionary mismatches between hosts and microbiota driven by cross-species microbial transmission. Similarly, although experiments in model systems have shown that disruption of microbiota can influence susceptibility to pathogens, the causal relationship between microbiota disruption and colonization resistance remains understudied in wildlife [157].

Three complementary approaches will be critical for advancing this area. First, low-cost shotgun metagenomic approaches [73] now enable characterization of microbiota functional potential, facilitating the generation of mechanistic hypotheses linking community composition to host traits. When feasible, coupling metagenomics with metabolomics would further strengthen inference by helping identify realized metabolic outputs of microbiota that may affect host physiology. Second, longitudinal field studies at the level of individuals or populations, combined with robust environmental and anthropogenic metadata, will be essential for linking microbiota dynamics to host outcomes; integration with statistical frameworks designed to infer causality, such as structural causal modeling, can further strengthen inference [158]. Third, experimental approaches, including microbiota transplantation of human-affected and unaffected wildlife microbiota into germ-free or gnotobiotic systems, provide powerful tools for testing causal relationships. However, experimental work in wildlife systems is often constrained by ethical and logistical considerations, necessitating the use of model organisms, non-invasive sampling, or carefully designed observational studies. In addition, model systems (e.g., germ-free mice) present limitations, including incomplete microbiota colonization and differences in host genotype-by-microbiota interactions relative to wildlife species [159]. Where possible, experiments conducted in phylogenetically proximate model systems may improve inference regarding causal effects of human-mediated microbiota disruption in wildlife.

### What are the microbial consequences of interactions among anthropogenic pressures?

Pollution, habitat transformation, and altered host contact networks can jointly restructure wildlife microbiota by altering both the selective environments experienced by microbes and the microbial source pool available for colonization. For example, pollutant exposure may select for resistant or detoxifying taxa, habitat transformation may change host physiology and microbiota dispersal within wildlife populations, and altered host contact networks may increase opportunities for microbes from humans, domestic animals, invasive species, or environmental reservoirs to colonize wildlife. In this framing, microbial invasion and cross-species transmission are not treated as independent causes of microbiota change, but as mechanisms that may be facilitated by anthropogenic changes in selection, dispersal, and host contact.

Disentangling these processes will be difficult because they often co-occur in real landscapes. Understanding their relative contributions will require integrative, often long-term study designs that pair sampling across anthropogenic exposure gradients with characterization of microbial source pools, pollutant concentrations, host ecology, and environmental conditions. Long-term field sampling platforms, such as the US National Science Foundation’s National Ecological Observatory Network, afford valuable opportunities for such studies, particularly when paired with targeted microbiota, metabolomic, and environmental sampling. Longitudinal sampling before and after disturbance events, such as habitat restoration, pollution remediation, or invasive species removal, will be particularly valuable for establishing temporal order and strengthening causal inference. Experimental approaches, including mesocosms, controlled exposure studies, and gnotobiotic systems, where appropriate, can complement these efforts by testing whether anthropogenic pressures alter colonization resistance and facilitate microbial establishment.

### What happens to understudied members of wildlife microbiota under anthropogenic influences?

Bacteria have dominated investigations of anthropogenic effects on wildlife microbiota, yet they represent only one dimension of a far more complex ecological network. Other microbial lineages, such as fungi, archaea, and bacteriophages, interact with hosts and bacterial communities through metabolic, immunological, and genetic pathways, affecting overall community structure and function. These groups are themselves responsive to anthropogenic pressures and can be transmitted across species boundaries. Although empirical data remain limited, emerging studies suggest that these components may be important in shaping microbiota responses to anthropogenic change. For example, phage–bacteria interactions can vary with habitat fragmentation [160], fungal communities shift with diet and disturbance [161], and archaea, which contribute to key metabolic processes such as methanogenesis [162], may respond to anthropogenic perturbation [163]. In addition, phage-mediated HGT has the potential to accelerate the spread of ecologically and clinically relevant traits, including AMR.

Despite their importance, these non-bacterial components remain underrepresented in current studies (S1 and S2 Tables), largely due to technical and analytical challenges, including low biomass, distinct cell structures that complicate extraction and amplification, biases in sequencing databases, and limited multi-kingdom analytical frameworks. Expanding the metacommunity perspective to incorporate these neglected components represents a key research priority and may fundamentally reshape our understanding of microbiota resilience and response to anthropogenic change.

## Conclusions

Anthropogenic changes reshape wildlife microbiota through interacting effects of environmental pollution, habitat transformation, and altered host contact networks. An important challenge now is to understand when and how these microbiota changes alter host physiology, immunity, fitness, and disease risk, and how these effects feed back on animal and human health. As the open questions discussed in this Unsolved Mystery are addressed, translating these insights into actionable strategies for conservation and human health will be an important next step. These applications may include: microbiota-informed interventions in captive or disturbed populations, such as restoring microbial exposure pathways; urban and landscape design that promotes beneficial microbial transmission within host species while limiting harmful spillover and spillback between wildlife and humans; and the use of microbiota features, such as shifts in diversity or AMR genes, as early warning signals of declining ecosystem health. Resolving these questions will provide a more comprehensive view of how human activities restructure host–microbe networks, alter eco-evolutionary trajectories, and shape outcomes for human, animal, and environmental health.

## Supporting information

S1 Table Selected primary research articles on the effects of pollution on wildlife gut microbiota. Studies were field-based or observational unless marked with † to indicate a laboratory experiment [164–166].
(DOCX)

S2 Table Selected primary research articles on the effects of habitat transformation on wildlife gut microbiota. Studies were field-based or observational unless marked with † to indicate a laboratory experiment [167,168].
(DOCX)

## Abbreviations

AMR: antimicrobial resistance
ARGs: antimicrobial resistance genes
HGT: horizontal gene transfer
