# Supplementary material for: How do anthropogenic changes affect wildlife microbiomes?
Source: PLoS Biol. 2026 Aug 26;24(8):e3003939. doi: 10.1371/journal.pbio.3003939 (PMC13514009; doi:10.1371/journal.pbio.3003939)
Supplement: Supplementary file 1 [file pbio.3003939.s001.docx]

## **S1 Table.** **Selected primary research articles on the effects of pollution on wildlife gut microbiota.** Studies were field-based or observational unless marked with † to indicate a laboratory experiment.

| **Perturbation** | **Host species** | **Location** | **Method** | **Outcome** |
| --- | --- | --- | --- | --- |
| Heavy metals (Cd, Pb, Cu) | Tree sparrow (*Passer montanus*) | China | 16S rRNA (V3–V4) | Decreased microbiota diversity, increased inter-individual variation, and enrichment of metal resistance functions [20]. |
| Metals/metalloids (As, Fe) | Earthworm (*Lumbricus rubellus*) | United Kingdom | 16S rRNA (V3–V5) | Community composition shifted along a contamination gradient, with loss of several taxa, including a symbiont [21]. |
| Cadmium^†^ | Earthworm (*Lumbricus terrestris*) | Germany | 16S rRNA (V4) | Exposure led to altered gut microbiota and increased abundance of heavy-metal-resistant bacterial taxa [22]. |
| Radionuclide exposure | Yellow-necked mouse (*Apodemus flavicollis*), wood mouse (*A. sylvaticus*), large and small Japanese field mice (*A. speciosus, A. argenteus*) | Ukraine and Japan | 16S rRNA (V4) | No effect on alpha diversity was observed, but species-specific responses of community composition were noted [23]. |
| 15 metals | Bank vole (*Myodes glareolus*) | Finland | 16S rRNA (V4) | Increased alpha diversity and shifts in community composition without change in dispersion; responses were taxon-specific [24]. |
| Diazinon^†^ | C57BL/6 mouse | United States (GA) | 16S rRNA (V4), meta-genomics | Altered community composition and metabolic profiles, with stronger effects in males [25]. |
| Glyphosate^†^ | C57BL/6J mouse | United States (IA) | Meta-genomics | Altered community composition, depleted beneficial bacteria, and increased inflammation [26]. |
| 18 pesticides^†^ | Male C57BL/6 mouse | United States (OH) | 16S rRNA (V4) | Altered microbial growth and metabolism and induced inflammation via lipid dysregulation [29]. |
| 140 environmental pollutants | Various small mammals | France | Mass spec-trometry | High chemical loads across sites indicated widespread contamination by legacy and current pesticides [30]. |
| Glyphosate^†^ | Honeybee (*Apis mellifera*) | United States (TX) | 16S rRNA (V4) | Altered community composition and increased susceptibility to infection by opportunistic pathogens [32]. |
| Chlorpyrifos^†^ | C57BL/6 and CD-1 (ICR) mice | China | 16S rRNA (V4–V5) | Disrupted gut barrier integrity, increased lipopolysaccharide translocation, and induced low-grade inflammation [33]. |
| Atrazine^†^ | Wasp (*Nasonia vitripennis*) | United States (MA) | 16S rRNA (V1–V4) | Altered community composition with transgenerational effects and increased resistance under repeated exposure [34]. |
| Imidacloprid^†^ | Honeybee (*Apis mellifera*) | United States (TX) | 16S rRNA (V4) | Decreased survival and increased pathogen susceptibility, but did not impact the microbiota [35]. |
| Cefoperazone^†^ | C57BL/6 WT mouse | United States (MI) | 16S rRNA (V3–V5) | Substantial changes to community composition and metabolome and increased susceptibility to *C. difficile* infection [37]. |
| Azithromycin, levofloxacin, cefpodoxime^†^ | Human (*Homo sapiens*) | United States (MO) | Meta-genomics | Acute loss of diversity; recovery occurred, but with altered composition, function, and increased AMR burden [38]. |
| Tylosin, amoxicillin^†^ | C57BL/6 mouse | United States (NY) | 16S rRNA (V4) | Early-life exposure caused persistent microbiota shifts and worsened colitis upon transfer to germ-free hosts [39]. |
| Minocycline, doxycycline^†^ | Male C57BL/6T mouse | United States (SC) | 16S rRNA (V3–V4) | Lasting shifts in composition, dysregulated liver metabolism, and increased adiposity [40]. |
| Sulfamethoxazole, oxytetracycline^†^ | Zebrafish (*Danio rerio*) | China | 16S rRNA (V3–V4) | Altered composition, increased inflammation, and impaired immune function and pathogen resistance [41]. |
| Tetracycline, erythromycin, sulfamethoxazole^†^ | Tadpole (*Rana omeimontis*) | China | 16S rRNA (full-length) | Reduced fitness, increased mortality and physiological abnormalities, with disrupted metabolome and altered composition [42]. |
| Antibiotic/ARG exposure | Golden snub-nosed monkey (*Rhinopithecus roxellana*) | China | 16S rRNA, meta-genomics | Increased prevalence of pathogenic and AMR genes with human disturbance, with disturbance-specific effects [43]. |
| 23 antibiotics in soil | Goitered gazelle (*Gazella subgutturosa*) | China | 16S rRNA (V3–V4) | Alpha diversity was lowest in gazelles in antibiotic-rich, vegetation-poor areas [44]. |
| Glyphosate or dicamba + ciprofloxacin or tetracycline^†^ | *Escherichia coli, Salmonella typhimurium* | New Zealand | In vitro culture + selection assays | *E. coli* evolved AMR up to 100,000× faster under combined exposure than under single treatments [50]. |
| Antibiotic/ARG exposure | Wild brown bear (*Ursus arctos*) | Sweden | Meta-genomics | Longitudinal data showed AMR load tracked human antibiotic use, increasing historically and declining with mitigation strategies [61]. |
| Wastewater/ agricultural exposure gradient | 408 wild birds and mammals | England | PCR (ARGs) | AMR prevalence varied across sites, time, and host groups, without consistent association with exposure risk [62]. |
| Antibiotic/ARG exposure | Western sandpiper (*Calidris mauri*), glaucous gull (*Larus hyperboreus*), Brent goose (*Branta bernicla*) | Siberia, Greenland | Culture-based AMR screening | Resistant and multidrug-resistant *E. coli* isolates were detected in the normal flora of Arctic birds [63]. |
| Antibiotic/ARG exposure | Nestlings of 16 wild avian species | Germany, Mongolia | PCR (ARGs) | Similar AMR prevalence across sites, suggesting migratory birds as vectors of clinically relevant AMR [64]. |
| Human proximity | 30 bird species | Europe and North America | WGS | Enhanced diversity and association with AMR strains in natural populations [66]. |
| Antibiotic/ARG exposure | Feral swine (*Sus scrofa*), coyote (*Canis latrans*), cattle (*Bos taurus*) | United States (FL) | 16S rRNA (V4), PCR (ARGs) | Wild animals harbored more AMR bacteria and ARGs than grazing cattle [164]. |
| Antibiotic/ARG exposure | Cattle (*Bos taurus*), sheep (*Ovis aries*), common vole (*Microtus arvalis*) | Germany | 16S rRNA (V4), MAGs | ARG profiles differed among hosts with limited sharing between species, indicating that transfer events between wildlife and livestock were infrequent [165]. |
| Antibiotic/ARG exposure | 75 wildlife species, 13 livestock species, humans | Kenya | Culture-based AMR screening | Urban wildlife carried high burdens of clinically relevant AMR, but phenotypic diversity was lower than in humans, livestock, or the environment [166]. |
| Antibiotic + metal co-exposure | Chicken manure, chicken feed, soil | China | 16S rRNA (V4–V5) | ARG abundance was enriched in manure and strongly associated with metals; mobile genetic elements (MGEs) co-occurred with ARGs across multiple antibiotic classes [68]. |
| Metal-contaminated soils | Soil | Ireland | 16S rRNA, PCR (ARGs, MGEs) | ARG and MGE abundance were strongly correlated, with metal-specific co-occurrence patterns [69]. |
| Metal (Zn, Cu, Cd, Cr) + oxytetracycline^†^ | Springtail (*Collembola*) | China | 16S rRNA, PCR (ARGs, MGEs) | Heavy metals induced ARG co-selection, though weaker than oxytetracycline; combined exposure produced strong synergistic effects [70]. |
| Mercury^†^ | Mummichog (*Fundulus heteroclitus*) | United States (NJ) | PCR (Hg resistance genes) | Hg resistance genes were >8× more abundant in contaminated sites and associated with multidrug resistance [71]. |

AMR, antimicrobial resistance; ARG, antimicrobial resistance gene; MAG, metagenome-assembled genome; MGE, mobile genetic element; PCR, polymerase chain reaction; WGS, whole-genome sequencing; WT, wild type.
