# Supplementary material for: How do anthropogenic changes affect wildlife microbiomes?
Source: PLoS Biol. 2026 Aug 26;24(8):e3003939. doi: 10.1371/journal.pbio.3003939 (PMC13514009; doi:10.1371/journal.pbio.3003939)
Supplement: Supplementary file 2 [file pbio.3003939.s002.docx]

## **S2 Table. Selected primary research articles on the effects of habitat transformation on wildlife gut microbiota.** Studies were field-based or observational unless marked with † to indicate a laboratory experiment.

| **Perturbation** | **Host species** | **Location** | **Method** | **Outcome** |
| --- | --- | --- | --- | --- |
| Anthropogenic disturbance gradient | Tome’s spiny rat *(Proechimys semispinosus*) | Panama | 16S rRNA (V4) | Species-specific responses, with reduced diversity, increased heterogeneity, and enrichment of human-associated taxa in disturbed environments [78,167]. |
| Urbanization gradient (land use) | Great tit (*Parus major*) | Poland | 16S rRNA (V3–V4) | Alpha diversity declined with impervious surface area and increased with tree cover; composition varied with urban features [79]. |
| Urban vs rural | Coyote (*Canis latrans*), crested anole lizard (*Anolis cristatellus*), white-crowned sparrow (*Zonotrichia leucophrys*) | Global | 16S rRNA (V4–V5) | Urban wildlife harbored human-associated microbes, with convergence of microbiota across urban populations [80]. |
| Captivity/ lifestyle disruption | Red-shanked douc (*Pygathrix nemaeus*), mantled howling monkey (*Alouatta palliata*) | Global | 16S rRNA (V4) | Loss of native microbes and convergence with the human microbiota in non-human primates [81]. |
| Protected land vs human settlement | Gray-brown mouse lemur (*Microcebus griseorufus*) | Madagascar | 16S rRNA (V4) | Disturbance reduced diversity and shifted composition toward pathogenic and metabolically altered communities [82]. |
| Urbanization, diet, age | American white ibis (*Eudocimus albus*) | United States (FL) | 16S rRNA (V3–V4) | Urban land cover and diet shaped microbiota; lower diversity was associated with increased *Salmonella* shedding [83]. |
| Urban vs rural | Beetle (*Carabus convexus*) | Hungary | 16S rRNA (V3–V4) | Lower diversity in urban beetles, with no compositional differences by habitat [84]. |
| Land-use change (agriculture/ logging) | Kuhl’s pipistrelle bat (*Pipistrellus kuhlii*) | Spain | 16S rRNA (full-length) | Alpha diversity varied across habitats, while community composition showed limited differentiation [89]. |
| Habitat type, fragmentation, captivity | Mexican black howler monkey (*Alouatta pigra*) | Mexico | 16S rRNA (V1–V3) | Microbiota diversity and composition tracked habitat and diet, with reduced diversity and functional capacity in suboptimal habitats [90]. |
| Urban vs rural | Black rat (*Rattus rattus*) | Turkey | 16S rRNA (V3–V4) | Rural rats had lower alpha diversity than urban rats [93]. |
| Captivity | Skywalker hoolock gibbon (*Hoolock tianxing*) | China | 16S rRNA (V3–V4) | Captive rearing was associated with lower alpha diversity and altered abundance of dominant taxa, with increased AMR-related functions relative to wild gibbons [94]. |
| Urbanization gradient | White-crowned sparrow (*Zonotrichia leucophrys*) | United States (CA) | 16S rRNA (V4) | Alpha and beta diversity were shaped by environmental factors and host traits, with urban communities driven by host morphology [95]. |
| Urban environmental factors | Anna’s hummingbird (*Calypte anna*) | United States (CA) | 16S rRNA (V1–V3) | Air quality was an important driver of microbial diversity [91]. |
| Land-use change | White-footed mouse (*Peromyscus leucopus*), deer mouse (*P. maniculatus*) | United States (MN) | 16S rRNA (full-length) | Agricultural and synanthropic habitats increased diversity and pathogen prevalence relative to forest habitats [92]. |
| Environment vs host physiology | Eastern gray squirrel (*Sciurus carolinensis*) | Canada | 16S rRNA (V3–V4) | Physiological measures better explained microbiota turnover than environment [110]. |
| Food limitation/ stress | Black howler monkey (*Alouatta pigra*) | Mexico | 16S rRNA (V4–V5) | Reduced food availability increased glucocorticoid levels and bacterial richness [109]. |
| Urban vs rural diet^†^ | House sparrow (*Passer domesticus*) | France | 16S rRNA (V5–V6) | Lower alpha diversity and altered beta diversity in urban birds, with enrichment of lipid metabolism and xenobiotic degradation pathways [101]. |
| Human-provisioned diet | American black bear (*Ursus americanus*) | United States (MI) | 16S rRNA (V4) | Human-provisioned food reduced microbial richness and phylogenetic diversity [102]. |
| Anthropogenic vs natural diet | Coyote (*Canis latrans*) | Canada | 16S rRNA (V4) | Urban coyotes consumed more anthropogenic food, had higher microbiota diversity, increased parasite prevalence, and poorer body condition [103]. |
| Warming^†^ | Common lizard (*Zootoca vivipara*) | France | 16S rRNA (V5–V6) | Warming (2–3°C) caused a 34% loss of diversity at the population level with potential negative impacts on host survival [105]. |
| Temperature shift^†^ | Western fence lizard (*Sceloporus occidentalis*) | United States (CA) | 16S rRNA (V4–V5) | Warming destabilized microbiota, reducing beneficial taxa and increasing potential pathogens; composition correlated with thermal tolerance [106]. |
| Warming + fragmentation^†^ | Common lizard (*Zootoca vivipara*) | France | 16S rRNA (V5–V6) | Warming (2°C) decreased microbiota diversity in isolated habitats, but connectivity offset or reversed these effects [108]. |
| Disturbance, connectivity | Northern cricket frog (*Acris crepitans*), American bullfrog (*Lithobates catesbeianus*) | United States (AL) | 16S rRNA (V4) | Greater disturbance increased dispersion, reduced similarity among sites, and was associated with lower Bd prevalence [113]. |
| Geographic barriers, disturbance | Milne-Edwards’s sifaka (*Propithecus edwardsi*) | Madagascar | 16S rRNA (V3–V5) | Geographic barriers structured beta diversity, while disturbance increased richness; geographic distance had no effect [96]. |
| Urbanization (multi-city) | Eastern gray squirrel (*Sciurus carolinensis*) | Canada | 16S rRNA (V4) | Phylogenetic patterns varied with coat color; agouti squirrels in urban environments showed greater phylogenetic beta dispersion than in rural or forest habitats [116]. |
| Anthropogenic disturbance gradient | Tome’s spiny rat (*Proechimys semispinosus*), common opossum (*Didelphis marsupialis*), gray four-eyed opossum (*Philander opossum*) | Panama | 16S rRNA (V4) | Species-specific responses across disturbance gradients, with reduced diversity and increased heterogeneity varying by host [98]. |
| Urbanization gradient | Small terrestrial mammals | France | 16S rRNA (V4) | Urban dwellers exhibited lower taxonomic diversity but higher functional diversity and distinct community composition relative to urban adapters [118]. |
| Urbanization (city size) | Striped field mouse (*Apodemus agrarius*) | Poland | 16S rRNA (V3–V4) | Ecological detachment did not consistently alter microbiota; green spaces were associated with retention of native gut communities [122]. |
| Urbanization gradient (land use) | Müller’s giant Sunda rat (*Sundamys muelleri*), black rat (*Rattus rattus*), Norway rat (*Rattus norvegicus*), Asian house shrew (*Suncus murinus*) | Borneo | 16S rRNA (V4) | Host identity primarily structured microbiota, with land use and spatial proximity influencing cross-species similarity [168]. |

AMR, antimicrobial resistance; Bd, *Batrachochytrium dendrobatidis*
